# Diversity and Antimicrobial Activity towards *Listeria* spp. and *Escherichia coli* among Lactic Acid Bacteria Isolated from Ready-to-Eat Seafood

**DOI:** 10.3390/foods10020271

**Published:** 2021-01-29

**Authors:** Jelena Stupar, Ingunn Grimsbo Holøymoen, Sunniva Hoel, Jørgen Lerfall, Turid Rustad, Anita Nordeng Jakobsen

**Affiliations:** Department of Biotechnology and Food Science, NTNU-Norwegian University of Science and Technology, NTNU, NO-7491 Trondheim, Norway; jelena.stupar@ntnu.no (J.S.); ingunn.g.holoymoen@ntnu.no (I.G.H.); sunniva.hoel@ntnu.no (S.H.); jorgen.lerfall@ntnu.no (J.L.); turid.rustad@ntnu.no (T.R.)

**Keywords:** biopreservation, ready-to-eat (RTE) seafood, *Carnobacterium*, *Leuconostoc*, *Listeria*, *E. coli*

## Abstract

Biopreservation is a food preservation technology using microorganisms and/or their inherent antimicrobial metabolites to inhibit undesirable microorganisms. The aim of the present study was to explore the diversity and antimicrobial activity of lactic acid bacteria (LAB) strains (*n* = 99) isolated from ready-to-eat (RTE) seafood (cold-smoked salmon (CSS), gravlax, and sushi) towards two strains of *Listeria monocytogenes* (CCUG 15527, F11), *Listeria innocua* (CCUG 15531) and *Escherichia coli* (CCUG 38079). The LAB strains were assigned to five different genera (*Carnobacterium* spp., *Lactobacillus* spp., *Leuconostoc* spp., *Weissella* spp., and *Enterococcus* sp.) by sequencing a 1150 bp stretch of the 16S rRNA gene. A significant association between the seafood source and the distribution of LAB genera was found (*p* < 0.001), of which *Leuconostoc* spp. were most prevalent in sushi and *Carnobacterium* sp. and *Lactobacillus* sp. were most frequently isolated from CSS and gravlax. Antimicrobial activity among the LAB was significantly affected by LAB genera (*F*= 117.91, *p* < 0.001, one-way ANOVA), product of origin (*F* = 3.47, *p* < 0.05), and target (*F* = 4.64, *p* = 0.003). LAB isolated from sushi demonstrated a significantly higher antimicrobial effect than LAB from CSS and gravlax (*p* < 0.05). In general, a significantly higher antimicrobial activity was found towards *Listeria* spp. than *E. coli* (*p* < 0.05). However, *Leuconostoc* spp. demonstrated similar antimicrobial effects towards *E. coli* and *Listeria* spp., except for *L. monocytogenes* F11 being more sensitive (*p* < 0.05). This study suggested that seafood-derived LAB strains could be selected for technological application in RTE seafood systems.

## 1. Introduction

The retail assortment of minimally and lightly processed seafood has increased tremendously and has been driven forward by consumer preferences for healthy, convenient, and ready-to-eat (RTE) products [1,2]. From a microbiological perspective, maintaining a good quality of these products is challenging and they might benefit from new approaches to seafood preservation. Seafood is perishable due to relatively high postmortem pH (>6), high water activity (*a*_w_), and high levels of soluble nitrogen compounds, supporting microbiological and biochemical activity [3]. Products in the RTE category can be eaten without further treatment to remove or reduce unwanted microorganisms before consumption, and it is important that the mild preservation methods be sufficiently effective against the pathogenic microorganisms that can be present in a given product [2,4,5,6].

Microbial seafood-borne diseases represent 10–20% of the reported foodborne outbreaks [7,8]. Examples of pathogenic bacteria commonly detected in seafood are *Escherichia coli, Aeromonas* spp., *Listeria monocytogenes, Salmonella* spp., *Staphylococcus aureus*, and *Vibrio parahaemolyticus* [9,10,11]. Of these, *L. monocytogenes* is among the most challenging pathogen for the seafood industry, regularly present in fresh and lightly processed seafood [4,12]. During 2010–2017, fish and fish products were the second-most common food categories contaminated by *L. monocytogenes*, approx. 16% of the total strong-evidence outbreaks reported. In 2018, the overall occurrence of *L. monocytogenes*-positive units in RTE fish was 2.5% at retail and production stages [7]. For instance, since 2015, Denmark, France, and Germany have been subject to a multi-country outbreak of *L. monocytogenes*, which is probably linked to cold-smoked salmon (CSS). Four deaths out of twelve confirmed cases of listeriosis had been reported by October 2018 [13]. Listeriosis is of especial concern for vulnerable groups, i.e., the elderly, pregnant women, infants, and others with weakened immune systems [14]. Following fecal or environmental contamination, *E. coli* can also be present in fresh fish, fresh bivalves, tuna paste, salted salmon roe, and processed seafood [15,16]. *E. coli* infections range from mild gastroenteritis to the most severe cases of hemolytic uremic syndrome (HUS) [16]. Of the total reported strong-evidence outbreaks caused by Shiga-toxin-producing *E. coli* (STEC) in European countries between 2010 and 2017, fish and fish products accounted for approx. 2% [7].

Biopreservation represents an innovative approach to preserving seafood that uses natural or added bacteria and/or their metabolites. The aim is to ensure safe products, increase the shelf life, and improve the hygienic quality of the products, with minimal effect on nutritional value and sensory properties. Lactic acid bacteria (LAB) are a group of bacteria shown to have the appropriate properties for this purpose, as they can produce different antimicrobial metabolites such as lactic acid, bacteriocins, diacetyl, hydrogen peroxide, and other organic acids [17,18,19,20]. In addition, many LAB have other properties that make them suitable for biopreservation of minimally and lightly processed seafood. First, they are considered safe for human consumption [17,18,19,20,21]. Further, many bioactive LAB isolated from seafood can grow at refrigerated temperature, and they are adaptable to processing and storage conditions commonly used for seafood, such as modified atmosphere packaging (MAP with elevated CO_2_ concentrations), vacuum packaging (VP), reduced pH, and added NaCl [21,22,23,24]. This is important, as biopreservation will be combined in a hurdle system together with, e.g., MAP or VP, and cold storage to attain an accumulative and sufficient effect on microbial growth without compromising sensory properties and levels of nutrients [25,26].

In seafood, biopreservation is mainly studied to control pathogens in lightly processed products (e.g., smoked or marinated salmon) [27,28,29,30,31,32,33], and many studies have successfully inhibited or reduced the growth of Gram-positive bacteria, e.g., *L. monocytogenes,* in various seafood products [29,31,32,33]. Previous studies have reported that *L. innocua* can be a suitable substitute for the pathogenic *L. monocytogenes* in challenge studies to make it possible to perform studies outside the laboratory, e.g., in pilot plants [34,35]. To our knowledge, there exists little information about inter- and intraspecies variation of *Listeria* spp. competitiveness towards a broad specter of antagonistic LAB [36]. Furthermore, LAB bacteriocins isolated from seafood are often shown to be inactive towards Gram-negative bacteria [8,37]. In addition, the beneficial properties do not apply to all LAB under all conditions. Some LAB strains can deteriorate seafood and lead to noticeable sensory changes [38]. Hence, it is important to carefully select specific strains for biopreservation that are both effective at low temperature and have no negative effects on the products of interest. Preserving products with certain sensory properties, such as CSS, that might mask any unfavorable effects following LAB growth is well documented [27,28,29,30,31,32,33]. Moreover, products like CSS have a relatively long shelf life, providing conditions for the LAB to outgrow inherent seafood bacteria. Although sparsely described in the literature [36,37,38,39], there is a potential for LAB biopreservation as a useful approach to preserving fresh fish. To our knowledge, no studies have yet examined the use of LAB biopreservation on fish intended for raw consumption, like sushi or sashimi. A clean sensory profile and a relatively short shelf life of such products prompt the need for potent LAB strains with a high growth rate at low temperature and the desired sensory effects on the product of interest. A first approach will be to investigate the diversity and antimicrobial effect of LAB adapted for growth in various RTE seafood products.

The aim of the present study was to explore the diversity and antimicrobial activity of LAB strains isolated from different RTE seafood (CSS, gravlax, and sushi) towards three bacterial species associated with lightly processed salmon products: *L. monocytogenes*, *L. innocua*, and *E. coli*. The screening was performed in a miniaturized salmon juice model system at 15 °C to identify psychrotrophic and target-inhibiting LAB strains that potentially can be used as bioprotective cultures in minimally or lightly processed salmon products.

## 2. Materials and Methods

### 2.1. Sampling, Isolation, and Quantification of LAB from Retail RTE Seafood

Strains of LAB were isolated from commercial VP CSS (*n* = 96 product packages) and VP gravlax (*n* = 48 packages) from four different producers purchased from retail stores in mid-Norway during a five-week period. The products were brought to the laboratory in chilled containers, kept at 4 °C after arrival, and analyzed within a few hours. Quantification and isolation of LAB were performed according to Nordic Committee on Food Analysis (NMKL) method no. 144 [40]. Isolates were confirmed as LAB by Gram staining and catalase testing before storage at −80 °C in Brain Heart Infusion (BHI) broth (Oxoid, Oslo, Norway) containing 20% glycerol (Sigma-Aldrich, Oslo, Norway) until further analysis. LAB previously isolated from commercial sushi (*n* = 58 sushi boxes) [41] were also included in the study. CSS is prepared from lightly salted salmon fillets subjected to traditional wood smoking at temperatures not exceeding 25–30 °C. The product characteristics are often based on the French standard NF V45-065; i.e., lipid < 18% (*w*/*w*), water content < 74%, a NaCl concentration of between 2.5% and 3.5% (*w*/*w*), and smoke treatment corresponding to 0.6 mg of phenol per 100 g of product [9]. Gravlax is usually prepared by curing salmon filets in a mixture of dry salt–sugar base and spices, with no pre-heating treatment or smoking process. It is mainly characterized by a salt content of 3–6% and by a pH of 5–6 [42]. Sushi meals typically consist of various species of raw (salmon, halibut, spring onion) and processed ingredients (cooked scampi, acidified cooked rice, wakame, ginger, wasabi). In total, 99 LAB strains were randomly selected for screening of bioprotective activity.

### 2.2. Temperature-Dependent Growth Assessment and Inoculum Preparation of Lactic Acid Bacteria

LAB strains were streaked twice on De Man, Rogosa, and Sharpe (MRS) agar (Oxoid, Oslo, Norway), incubated anaerobically in anaerobic incubation containers with GasPak EZ Anaerobe container system sachets w/indicator (BD, Oslo, Norway) at 25 °C for 2–5 days, transferred into MRS broth (Oxoid, Oslo, Norway), and cultivated at 15 °C in 15 mL tubes with MRS broth to assess their ability to proliferate at 15 °C. Cell growth was monitored until the cultures reach the stationary phase (approx. 120 h of cultivation) by measuring the optical density at 600 nm (OD_600_; Shimadzu UV 1800; Shimadzu, Duisburg, Germany) after proper dilution. For preparation of LAB inoculums for the coculture assay, a colony was transferred from the MRS agar plate into MRS broth for overnight incubation at 15 °C to adapt to the lower temperature in the inhibitory assay. The cultures were standardized to a concentration of 10^8^ CFU/mL by proper dilution in MRS broth to obtain an optical density of 0.22 at 600 nm (Shimadzu UV 1800; Shimadzu, Duisburg, Germany).

### 2.3. Preparation of Target Strains

The target organisms *L. innocua* (CCUG 15531), *L. monocytogenes* (CCUG 15527), an environmental strain of *L. monocytogenes* (F11, collected from a Salmon deheader), and *E. coli* (CCUG 38079) were streaked on BHI agar and incubated overnight at 37 °C. Fresh cultures were then transferred into BHI broth and incubated overnight at 15 °C for temperature adaptation. The cultures were standardized to an optical density of 0.1 at 600 nm (Shimadzu UV 1800, Shimadzu, Duisburg, Germany) and further diluted in BHI broth to obtain a concentration of 10^4^ CFU/mL.

### 2.4. Salmon Juice Preparation

Enriched salmon juice was used as a growth medium for cocultivation of LAB and target strains. Although not directly comparable to the conditions in a salmon fillet, salmon juice was chosen as a growth medium to mimic the nutritional composition of the fish. The juice was prepared from fresh salmon loins obtained from a local retailer, as described by Wiernasz, Cornet, Cardinal, Pilet, Passerini, and Leroi [27]. In brief, 500 g of salmon loin was blended with 1 L of distilled water, boiled for 2 min, filtered through a 185 mm folding filter (Schleicher & Schuell, Dassel, Germany), and sterilized at 100 °C for 30 min. Sterile salmon juice was stored at −40 °C. Before use, 90 mL of salmon juice was supplemented with 10 mL of 1 M K_2_HPO_4_/KH_2_PO_4_ buffer solution (Merck, Darmstadt, Germany) at pH 6.7, 1 g of D-glucose (Merck, Oslo, Norway), and 1.5 g of NaCl (VWR, Leuven, Belgium) to prevent growth inhibitory conditions in the model system. After enrichment, the medium was sterilized through a 0.45 μm syringe filter (VWR, Oslo, Norway).

### 2.5. Inhibitory Assay on Target Organisms in 96-Well Plates

A coculture assay was used to test each LAB strain for the ability to inhibit growth of the target strains, as described by Wiernasz, Cornet, Cardinal, Pilet, Passerini, and Leroi [27]. In brief, cocultures were performed in 96-well plates containing 196 µL of enriched salmon juice and 2 µL of the standardized inoculums. Thus, initial concentrations in the assay were 10^6^ CFU/mL for LAB and 10^2^ CFU/mL for targets. A growth assay temperature of 15 °C was chosen to ensure adequate growth rates of all LAB strains as well as target strains. Un-inoculated enriched salmon juice was used as a negative control, whereas cultures of LAB monocultures were included to confer growth in the salmon juice medium. Target monocultures were included as a reference for quantification of growth inhibition in coculture. For bacterial quantification, a serial dilution was performed in peptone water (1 g/L of peptone (Oxoid, Oslo, Norway) and 8.5 g/L of NaCl (VWR, Leuven, Belgium), followed by micro-spotting of 5 µL of each dilution on target-selective agars: Brilliance Listeria Agar (BLA) (Oxoid, Oslo, Norway) with Brilliance Listeria Selective Supplement (Oxoid, Oslo, Norway, incubated at 37 °C for 24 h) for *Listeria* spp. and Violet Red Bile Agar (VRBA) (Oxoid, Oslo, Norway, incubated at 37 °C for 24 h) for *E. coli*. The target strains were also quantified on BHI agar for detection of possibly unculturable cells on the selective agars. LAB was quantified on MRS agar (anaerobically in anaerobic incubation containers with GasPak EZ Anaerobe container system sachets w/indicator (BD, Oslo, Norway) at 25 °C for 2 days).

LAB monocultures and the cocultures were performed with four biological parallels in 96-well plates, while eight biological parallels were conducted for all target monocultures. All experiments showing growth inhibition were repeated twice in biologically independent replicates.

### 2.6. Molecular Identification and Phylogenetic Analysis of Lactic Acid Bacteria

Cell pellets for DNA extraction were harvested from 1 mL of LAB culture centrifuged at 8000× *g* for 5 min. Total genomic DNA was extracted using the protocol for Gram-positive bacteria in the DNeasy Blood and Tissue Kit (Qiagen, Oslo, Norway). PCR targeting 16S rRNA was performed with 5 μL of extracted DNA and 45 μL of master mix (1× PCR buffer (1.5 mM MgCl_2_), 200 µM of each nucleotide, 0.2 µM of each primer, and 2.5 U Taq polymerase (Qiagen)). An approx. 1150 bp sequence of the bacterial 16S rRNA gene was amplified with the primers 338F (5′-ACTCCTACGGGAGGCAGCAG-3′) [43] and 1492R (5′-TACGGYTACCTTGTTACGACT-3′) [44] (Sigma Aldrich, Oslo, Norway) using a Thermal Doppio PCR instrument (VWR, Oslo, Norway) and the following thermal profile: initial denaturation and heat activation for the polymerase at 95 °C for 15 min, followed by 30 cycles of 95 °C for 1 min, annealing at 58 °C for 30 s and polymerization at 72 °C for 1 min, followed by a final extension at 72 °C for 5 min. The size of the PCR products was confirmed by a standard agarose gel electrophoresis. The PCR products were purified using a GeneJET PCR Purification kit (Thermo Fisher Scientific, Oslo, Norway), and Eurofins Genomics (Ebersberg, Germany) performed the sequencing. The obtained DNA sequences were assigned to their genus using the RDP (Ribosomal Database Project) Classifier [45]. The sequences were also compared with available sequences in the NCBI GenBank database using nucleotide BLAST [46] to obtain a putative species identification. Multiple-sequence alignment was performed using ClustalW, integrated in the MEGA X software [47]. The 16S rRNA gene sequences from all isolates (*n* = 90) and type strain sequences for a selection of LAB retrieved from the NCBI GenBank database were included in the alignment. A phylogenetic tree was constructed using the neighbor-joining method [48], with 1000 bootstrap replicates to assess the tree topology robustness, and evolutionary distances (nucleotide substitutions per site) were computed using the maximum composite likelihood method [49].

### 2.7. Data Treatment and Statistical Analysis

Statistical analysis was performed on log-transformed data. One-way ANOVA tests were carried out using Tukey HSD (honestly significant difference) procedures to derive statistical differences (*p* < 0.05) of antimicrobial activity among the LAB strains based on the factors LAB genera, product, and target. An independent-samples *t*-test was used to compare target growth on selective and general media.

The chi-square test of independence was applied to test for differences in the distribution of LAB genera among the seafood sources (CSS, sushi, and gravlax). The Statistical Package for the Social Science (SPSS, version 26, IBM, Armonk, NY, USA) was used for all analyses.

The level of growth inhibition was calculated by the following formula:

(1) $$\Delta \log\mathrm{CFU}/\mathrm{mL}\;=\overset{-}{x}\;\log\;\mathrm{CFU}/\mathrm{mL}\;\mathrm{target}\;\mathrm{monoculture}-x\;\log\;\mathrm{CFU}/\mathrm{mL}\;\mathrm{target}\;\mathrm{in}\;\mathrm{coculture}\;\mathrm{with}\;\mathrm{LAB}\;$$

where Δ log CFU/mL represents the inhibition level, i.e., difference between average concentration (log CFU/mL) of target cells grown in monoculture (*n* = 8) and concentration (log CFU/mL) of target obtained in coculture with LAB (*n* = 4) (incubation conditions stated in Section 2.5). Results are given in log CFU/mL with standard deviation.

## 3. Results

### 3.1. Isolation and Identification of LAB from Retail RTE Seafood

**Figure 1:**
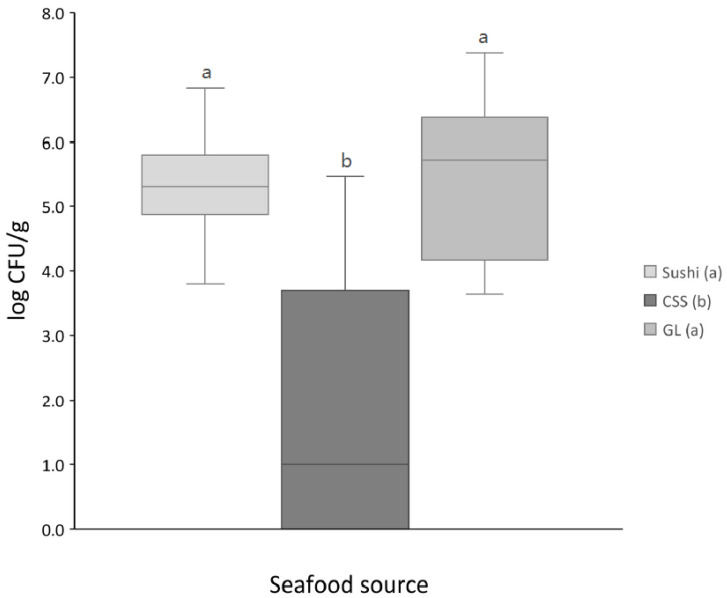
Box plot of the log-transformed LAB counts from cold-smoked salmon (CSS, *n* = 96), gravlax (GL, *n* = 44), and sushi (*n* = 57). The solid horizontal line inside the box indicates the median, and whiskers indicate the range. Boxes with different letters have means that are significantly different (*p* < 0.05).

The mean LAB counts in sushi and gravlax products were 5.3 ± 0.8 and 4.7 ± 2.6 log CFU/g, respectively, and significantly higher (*p* < 0.05) than in CSS with 1.9 ± 2.0 log CFU/g (Figure 1). The results revealed high variations in the LAB counts in CSS and gravlax products, ranging from not detected to above 7 log CFU/g in the different packages.

In total, 99 LAB strains isolated from CSS (*n* = 33), sushi (*n* = 44), and gravlax (*n* = 22) were included in the study. Of these, 92 isolates were able to proliferate and reach the stationary phase within 96 h of cultivation at 15 °C in MRS broth. The remaining isolates were excluded from further analysis. High-quality sequence reads (676–1145 nt) were obtained for 90 strains, which were assigned to five different genera (identity scores 100%, RDP Classifier) (Table 1). A chi-square test of independence showed that there was a significant association between the seafood source and the distribution of LAB genera (*X*^2^ (8, *N* = 90) = 41.5, *p* < 0.001). For instance, *Leuconostoc* spp. were most prevalent in sushi, whereas *Carnobacterium* spp. were most frequently isolated from CSS.

**Figure 2:**
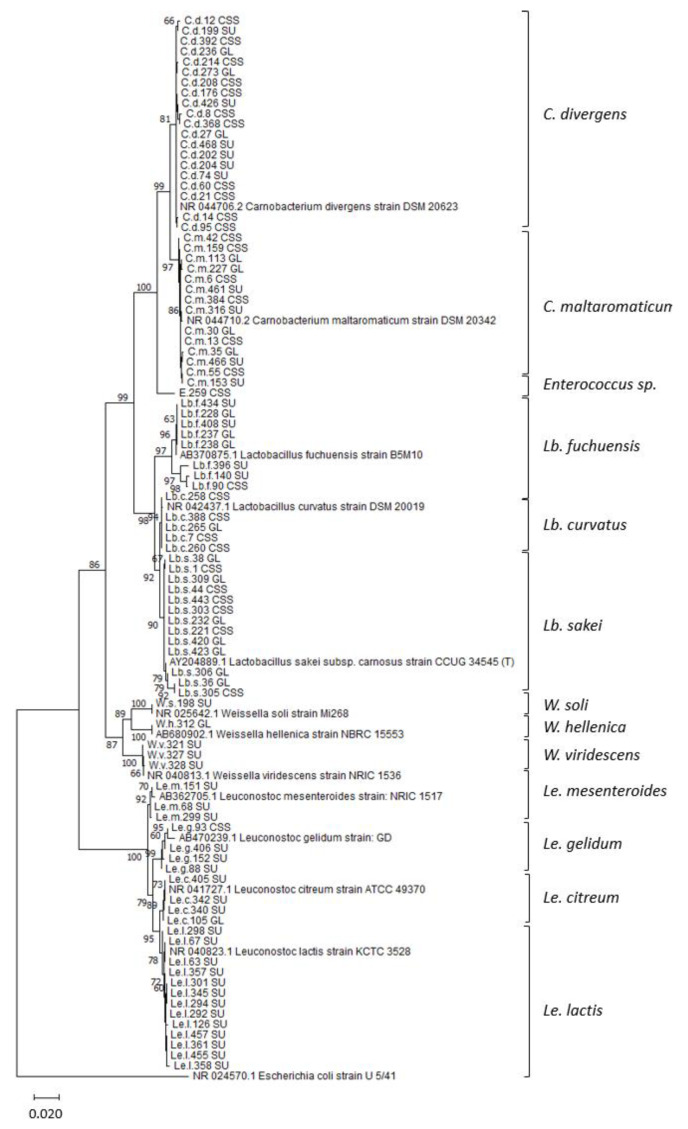
Neighbor-joining tree based on partial 16S rRNA gene sequences of lactic acid bacteria isolated from sushi (SU), cold-smoked salmon (CSS), and gravlax (GL). The evolutionary distances were computed using the maximum composite likelihood method. Reference sequences retrieved from the GenBank database are indicated with gene bank accession numbers. The percentage of 1000 bootstrap replicates is shown next to the branches (only values >50% are shown). The scale bar indicates an evolutionary distance of 0.02 nucleotide substitutions per site. The analysis included 103 sequences and a total of 1124 positions in the final dataset. *Escherichia coli* (acc. no NR024570) was used as an outgroup.

To identify the LAB isolates at the species level, a BLAST search [46] was performed. Concordance in the best matches was observed, and the similarity scores were ≥99% in all cases. The only exception was the *Enterococcus* strain, which could not be assigned to any species due to a 100% sequence similarity to a number of different *Enterococcus* species. A phylogenetic tree (Figure 2) based on the partial 16S rRNA gene sequences from evolutionary distances using the neighbor-joining method was constructed to confer clustering of sequences that had been assigned to the same species. The overall mean sequence divergence of in the total dataset was 2.7%, ranging from 0.8% for the *Carnobacterium* sequences to 3% for the *Weissella* sequences. The 34 *Carnobacterium* isolates clustered into two distinct branches in the phylogenetic tree, of which 59% of the carnobacteria were identified as *Carnobacterium divergens* and 41% as *Carnobacterium maltaromaticum*. Both species were present in all three salmon products. The 26 LAB isolates assigned to the genus *Lactobacillus* were further identified as *L. sakei* (50% of the lactobacilli), *Lactobacillus curvatus* (19%), and *Lactobacillus fuchuensis* (31%), and all *Lactobacillus* species were present in all salmon products. *Leuconostoc* spp., mostly isolated from sushi, were identified as *Leuconostoc lactis* (54%), *Leuconostoc citreum* (17%), *Leuconostoc gelidum* (17%), and *Leuconostoc mesenteroides* (12%). The species diversity was relatively high among the five isolates belonging to the genus *Weissella*, of which three isolates were assigned to the species *Weissella viridenscens* and the remaining to *Weissella soli* and *Weissella hellenica*.

### 3.2. Antimicrobial Activity of LAB towards Target Strains

The antimicrobial activity of the 92 LAB strains towards two strains of *L. monocytogenes*, one strain of *L. innocua*, and *E. coli* was examined in a coculture 96-well plate setup in enriched salmon juice at 15 °C. Quantification of target monocultures was performed in parallel on selective and general media to confirm that there was no growth inhibition on selective media. *E. coli* was quantified on VRBA (9.08 ± 0.44 log CFU/mL) and BHI agar (9.30 ± 0.29 log CFU/mL), *L. monocytogenes* (CCUG 15527) on BLA (9.09 ± 0.40 CFU/mL) and BHI agar (9.35 ± 0.51 log CFU/mL), *L. monocytogenes* (F11) on BLA (9.39 ± 0.53 log CFU/mL) and BHI agar (9.40 ± 0.39 log CFU/mL), and *L. innocua* on BLA (9.38 ± 0.42 log CFU/mL) and BHI agar (9.34 ± 0.37 log CFU/mL). Although the mean log CFU/g of *E. coli* and *L. monocytogenes* CCUG 15527 on selective and general media was statistically different (*p* < 0.001), the comparison of bacterial counts does not, from a biological perspective, indicate any growth inhibition on selective media.

The final cell concentrations of LAB monocultures ranged from 8 to 10 log CFU/mL (*n* = 4, SD < 2%) and verified that the bacteria were able to grow in the fish juice. We can therefore assume that the target growth inhibition detected in the cocultures was due to the effect of LAB, not the medium.

Overall, the antimicrobial activity was significantly affected by the bacterial genus (LAB) (*F* = 117.91, *p* < 0.001), product of origin (*F* = 3.47, *p* < 0.05), and target (*F* = 4.64, *p* = 0.003). Of the five LAB genera, *Lactobacillus* sp. demonstrated a significantly lower effect than the others (*p* < 0.05). Between *Leuconostoc* sp., *Weissella* sp., and *Carnobacterium* sp., no significant differences were observed. Further, LAB isolated from sushi had a significantly higher antimicrobial effect than LAB from CSS and gravlax (*p* < 0.05).

Among all LAB, there were no significant differences in th einhibition level against the three *Listeria* strains (*p* = 0.30). However, the effect towards *L. monocytogenes* sp. was numerically higher than for *L. innocua*. The antimicrobial effect among the LAB strains was found to be significantly higher towards *Listeria* sp. than *E. coli* (*p* < 0.05).

Further, interesting results were found among *Leuconostoc* sp., with no significant difference in effect against *E. coli,* the *L. monocytogenes* reference strain (CCUG 15527), and *L. innocua* (*p* = 0.86), but a higher effect towards the *L. monocytogenes* environmental strain (F11) compared to *E. coli* and *L. innocua* (*p* < 0.05), and at a significance level of 10% against the *L. monocytogenes* reference strain. At a significance level of 10%, *Weissella* sp. was more active against *L. innocua* than against *L. monocytogenes* sp. (*p* < 0.1), while *Carnobacterium* sp. was more active against *L. monocytogenes* sp. than against *L. innocua* (*p* < 0.1). Although *Lactobacillus* sp., in general, had a low antimicrobial effect towards all targets (below 4.4 log CFU/mL reduction), a significantly higher effect against the *L. monocytogenes* reference strain than against the other three targets (*p* < 0.05) was observed.

**Figure 3:**
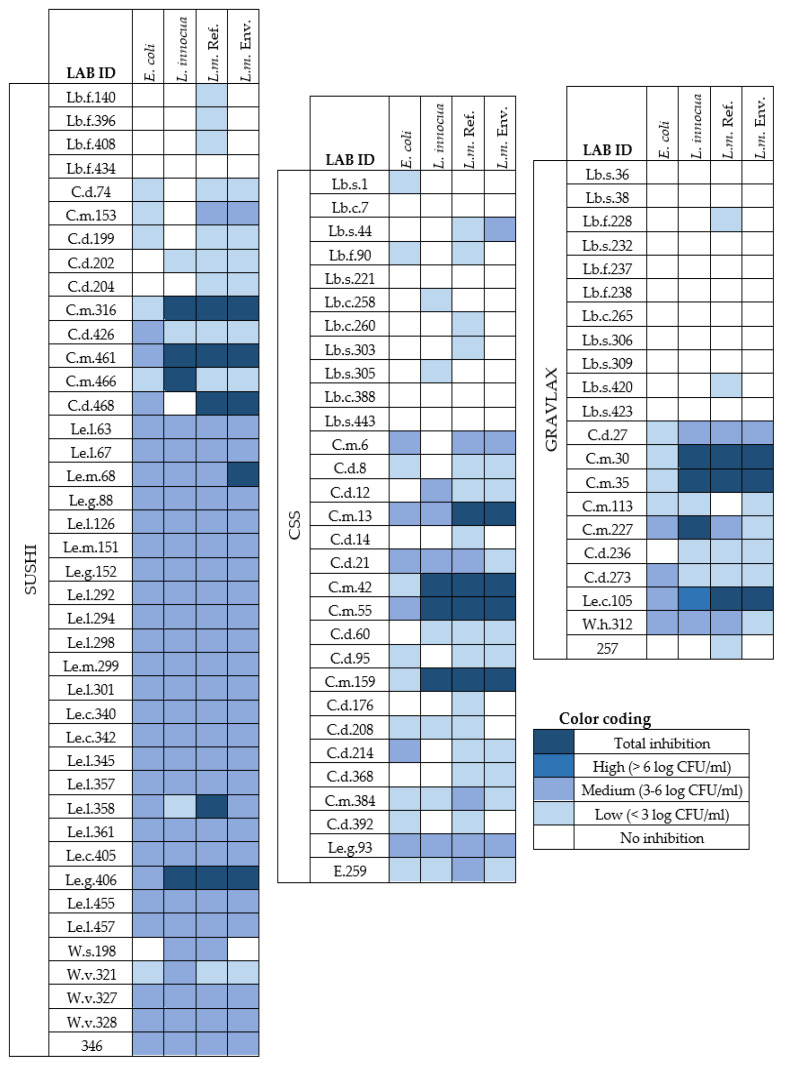
Growth inhibitory effect of LAB isolated from sushi (*n* = 41), CSS (*n* = 30), and gravlax (*n* = 21) against *L. innocua*, the *L. monocytogenes* (L.m.) reference strain, the L. monocytogenes environmental strain, and E. coli. Inhibition levels are given in color codes, as shown, based on Δlog CFU/mL, i.e., the difference between the average concentration (log CFU/mL) of the target monoculture (*n* = 8) and the concentration (log CFU/mL) of the target obtained in coculture with LAB (*n* = 4). C.d.: Carnobacterium divergens; C.m.: C. maltaromaticum; Lb.s.: Lactobacillus sakei; Lb.f.: Lb. fuchuensis; Lb.c.: Lb. curvatus; Le.c.: Leuconostoc citreum; Le.g.: L. gelidum; Le.l.: L. lactis; Le.m.: L. mesenteroides; W.h.: Weissella hellenica; W.s.: W. soli; W.v.: W. viridenscens; E.p.: Enterococcus pseudoavium. Strains with only a number were not identified.

For a biological interpretation of the data, we chose to categorize the inhibition levels into five categories: no effect, low effect (<3 log CFU/mL reduction), medium effect (3–6 log CFU/mL reduction), high effect (>6 log CFU/mL reduction), and total inhibition (no detectable growth of target organism). Figure 3 summarizes the inhibition level of LAB (cf. Equation (1)) isolated from CSS, gravlax, and sushi against targets based on these categories. The bacterial concentrations of all LAB–target cocultures are provided in Supplementary Table S1.

#### 3.2.1. LAB Isolated from Sushi

More than 80% of the LAB isolates from sushi (33 out of 41) displayed a medium to total inhibitory effect against at least one target (Figure 3). Two *C. maltaromaticum* strains (C.m.316 and C.m.461) and one strain identified as *L. gelidum* (Le.g.406) exhibited total inhibition of the three *Listeria* targets, while low (C.m.316) or medium (C.m.461, Le.g.406) activity was observed towards *E. coli*.

All isolates identified as *Leuconostoc* showed medium antimicrobial activity towards all targets. The only exception was *Le. lactic* strain Le.l.358, with low activity against *L. innocua.* The same strain (Le.l.358) showed medium activity towards *E. coli* and the environmental *L. monocytogenes* strain, while total inhibition was observed against the other strain of *L. monocytogenes.* Overall, a 3–5 log CFU/mL reduction in *E. coli* was observed in coculture with all *Leuconostoc* strains from sushi.

Only minor variations in the inhibitory activity against *Listeria* sp. were observed among the *Carnobacterium* strains, except for isolate C.m.466, displaying total inhibition of *L. innocua* and low antimicrobial activity against both *L. monocytogenes* targets, and C.d.468, displaying a contrary effect, with low antimicrobial activity against *L. innocua* and total inhibition of *L. monocytogenes* sp.

Two of the four isolates belonging to the genus *Weissella* displayed medium activity against all targets. None or low antimicrobial activity towards the targets studied was observed among the *Lactobacillus* isolates from sushi.

#### 3.2.2. LAB Isolated from Cold-Smoked Salmon

Of the CSS LAB isolates, 40% (12 of 30) displayed a medium to total inhibitory effect against at least one target. The strains with the highest anti-listerial activity were identified as *Carnobacterium* spp., of which 23.5% showed medium to total inhibition towards all three *Listeria* sp. tested. Among these, three *C. maltaromaticum* strains (C.m.42, C.m.55, and C.m.159) were the most active, displaying total inhibition against all *Listeria* targets. *C. maltaromaticum* strain C.m.13 displayed total inhibition of both strains of *L. monocytogenes* but only medium activity towards *L. innocua* (5.6 log CFU/mL reduction). Interestingly, several strains of *C. maltaromaticum* (C.m.55, C.m.6, and C.m.13) and *C. divergens* (C.d.21) were medium active towards *E. coli*, with 3.1–4.0 log CFU/mL reduction.

The only strain from the genus *Leuconostoc*, *Le. gelidum* (Le.g.93), displayed medium activity (approximately 4 log CFU/mL reduction) towards all target strains. The weakest inhibitory effects among the LAB isolates from CSS were found in the *Lactobacillus* group, displaying no significant or low (0–1.7 log CFU/mL) inhibition towards all targets tested. The only exception was the *Lb. sakei* strain Lb.s.44, demonstrating low to medium activity (0.4–4.3 log CFU/mL reduction) against both strains of *L. monocytogenes*.

#### 3.2.3. LAB Isolated from Gravlax

More than 30% of the LAB isolates from gravlax (7 of 21) displayed medium to total inhibition of at least one target strain. Two strains of *C. maltaromaticum* (C.m.30 and C.m.35) displayed total inhibition against all *Listeria* targets, while another strain of the same species (C.m.227) showed total inhibition only against *L. innocua.*

The only strain identified as *Leuconostoc* sp., *Le. citreum* (Le.c.105), displayed total inhibition against both *L. monocytogenes* strains and high inhibition with more than 7 log CFU/mL reduction in *L. innocua.*

Several strains (Le.c.105, C.m.227, C.d.273, W.h.312), demonstrated medium activity against *E. coli* (>5 log CFU/mL reduction). All *Lactobacillus* strains isolated from gravlax exhibited none or low antimicrobial activity against all targets.

## 4. Discussion

In the present study, the bioprotective potential of LAB isolated from RTE seafood as future candidates for biopreservation of lightly preserved seafood products was assessed. To our knowledge, LAB isolated from sushi and gravlax have never been tested for use in biopreservation. It is likely to believe that LAB from different sources can have different inhibition activities in a given product, as they are adapted to different environments. LAB strains originating from RTE seafood can be especially applicable as preservative cultures in similar products due to environmental adaptation [50,51,52].

Variations in LAB counts and distribution of LAB genera of the RTE salmon products analyzed were observed, and the mean LAB counts in sushi and gravlax were significantly higher than in CSS. In general, LAB are not a major part of the normal microbiota of fish flesh, due to a relatively high postmortem pH, low sugar content, and high levels of soluble nitrogen compounds [21]. However, if the fish products are lightly preserved, e.g., by addition of NaCl, LAB can become predominant [21]. Gram-negative bacteria are generally inhibited under these conditions, whereas many LAB will thrive as they are psychrotrophic, salt tolerant (up to 8–10% salt), and able to catabolize arginine [21,53]. The salt content of CSS and gravlax is normally from 2.5 to 3.5% [38] and 3 to 6% [42], respectively. The salt content of sushi varies greatly, depending on the ingredients [54], and was reported to be 1.2% (*w*/*w*) in the boiled, acidified rice in retail sushi [55].

LAB are generally less competitive in fresh fish [21,56]; however, in complex products such as sushi, composed of fresh raw fish and a variety of ingredients, LAB can be a dominant part of the microbiota [41], probably selected by the low pH in the acidified sushi rice (pH < 4.6) [21,57]. The pH of sushi is low compared to CSS with pH 6.0–6.3 [38,58] and GL with pH 5–6 [42].These factors suggest that the LAB included in our study are adapted to different environments, and a significant association between the seafood source and the distribution of LAB genera was confirmed. Our screening of 92 LAB isolates representing 5 genera and 13 species allowed us to assess intraspecies biodiversity based on the source of isolation. *Leuconostoc* spp. were the most prevalent in sushi, whereas *Carnobacterium* spp. and *Lactobacillus* spp. were the most frequently isolated LAB from CSS and gravlax. *Carnobacterium* sp. (especially *C. maltaromaticum* and *C. divergens*), species of *Lactobacillus* (especially *Lb. sakei* and *Lb. curvatus*), *Leuconostoc* sp., and *Lactococcus* spp. were the most frequently LAB isolated from cold-smoked salmon and gravlax products reported by others [17,21,28,59]. Only one *Enterococcus* strain was detected in CSS. The origin of this strain is of interest, since enterococci are recognized as pathogenic strains or taste and flavor contributors of fermented food (starter cultures) [60]. Strains from this genus are isolated from aquatic environments and sewages but also from RTE products [17]. *Weissella* sp. is commonly found in fermented seafood [61] and traditional smoked and sun-dried fish products [62]. Further, the presence of *Weissella* spp. is associated with gravlax [28] and sushi [63], in agreement with the present study. Interestingly, in our study, *Weissella* strains were detected in sushi and gravlax but not in CSS. Hence, the isolation of *Weissella* spp. could be attributed to products containing ingredients of vegetable origin.

A growth assay temperature of 15 °C was chosen to ensure adequate growth rates of all LAB strains as well as target strains. The sub-optimal temperature allowed for exclusion of strains that could not proliferate at 15 °C (seven strains), and at the same time a rapid screening of the growth inhibitory properties of a large number of strains. The applied assay temperature was higher than the expected storage temperature for seafood products. Thus, the LAB strains that are selected for further characterization to become candidates for biopreservation of cold-stored, minimally lightly processed RTE seafood products must be tested at 4 °C or lower [64]. The salmon juice that was used as a growth medium had a salt content of 1.5% and a pH of 6.7, which are probably not the optimum levels for all the LAB strains. However, the control samples with LAB monocultures and target monocultures demonstrated that all targets and the 92 LAB strains were able to proliferate and reach the stationary phase in the fish juice with the conditions used in the inhibitory assay (96 h at 15 °C), indicating that environmental factors most likely are not limiting factors in our setup. The sushi isolates’ ability to grow in salmon juice indicates their potential as preservative cultures in cold-stored salmon products, even if they are of vegetable origin.

For the purpose of adding bioprotective strains of LAB to food, accurate strain identification is required. Although widely used for bacterial classification and identification, the 16S rRNA gene has some limitations regarding taxonomic resolution for distinct groups of bacteria [65,66]. In the present study, a total of 90 LAB strains were identified at the genus level and at the putative species level using a relative long fragment of 16S rRNA containing the variable regions V3 to V9 (>1000 nt for most of the sequences). However, the overall sequence similarity was 97.3%, and the intragenus sequence similarities were higher for all genera (98.6–99.2%), except *Weissella* spp. The putative species identification was supported by the constructed phylogenetic tree that showed clustering of sequences assigned to the same species in well-separate branches with bootstrap values ≥78%. However, the species assignations presented here must be interpreted with caution. A better taxonomic resolution was observed when sequencing fragments containing the V1 and V2 regions of the 16S rRNA gene [18,67,68] and should be included in future strain characterization.

It is generally accepted that LAB and/or their bacteriocins isolated from seafood have the potential to inhibit or delay the growth of *L. monocytogenes* in seafood products, such as CSS and gravlax [27,28,29,30,31,32,33]. In a previous study, it was observed that LAB were able to grow to high concentrations in sushi [57], and in the present study, LAB from sushi had a significantly higher antimicrobial effect than LAB from CSS and gravlax (*p* < 0.05). Of particular importance, this suggest the potential of LAB in biopreservation of RTE fish products that are not salted, fermented, or cold smoked. As LAB strains can have various effects in different products, one must carefully select bacteria that are suitable for the exact product [17,21]. It is important to note that environmental factors, e.g., NaCl, pH, and temperature, affect bacteriocin production of LAB, but also susceptibility of target strains [69].

For application in food biopreservation, strains displaying broad antimicrobial activity towards both Gram-negative and Gram-positive targets are of interest. Therefore, both *Listeria* sp. and *E. coli* were included as targets in our study. The most promising candidates for biopreservation were found among strains of *Carnobacterium* and *Leuconostoc*. Several strains from these genera demonstrated a broad antimicrobial effect towards both Gram-negative and Gram-positive targets by total inhibition of *Listeria* sp. and medium activity against *E. coli*. The inhibition mechanisms were not examined in this study but were previously discussed by Leisner, Laursen, Prévost, Drider, and Dalgaard [69].

Furthermore, to explore the inter- and intraspecies variation of *Listeria* sp. competitiveness towards antagonistic LAB and the potential variations between environmental and reference (culture collection) strains of *L. monocytogenes*, three *Listeria* strains were included in the screening of LAB antimicrobial activity. Our results are not conclusive regarding inhibition levels towards *Listeria* spp. Although an overall difference in the inhibition level against the three *Listeria* strains was not found (*p* = 0.30), a tendency of *L. innocua* being more resistant to the antimicrobial activity of LAB than the *L. monocytogenes* strains was observed. When comparing the inhibition level of *L. monocytogenes* sp. and *L. innocua* individually, a subset of strains (C.m.6, C.m.13, C.m.384, C.m.153, C.d.468, and Le.l.358) showed higher inhibition activity towards *L. monocytogenes* than *L. innocua* (Figure 3). Only two strains (C.m.227 and C.m.466) were significantly more active against *L. innocua* than *L. monocytogenes.* Based on this, further studies are needed to clarify whether *L. innocua* can be used as a substitute for *L. monocytogenes* in challenge studies, as previously indicated by Scott, Swanson, Freier, Pruett Jr., Sveum, Hall, Smoot, and Brown [34] and Vaz-Velho, Fonseca, Silva, and Gibbs [35].

No overall difference in sensitivity was observed between the reference strain and the environmental strain of *L. monocytogenes*, but differences were observed among a subset of 23 LAB isolates that were more active against the *L. monocytogenes* reference strain compared to the environmental strain (except Le.m.68, Lb.s.44, and C.m.113). Also Brillet, Pilet, Prevost, Bouttefroy, and Leroi [36] found differences in sensitivity among a variety of *L. monocytogenes*. However, in that study only three LAB were tested, all belonging to *Carnobacterium*.

Moreover, intraspecies differences in antimicrobial activity were observed within the *Carnobacterium* genus, between strains from all three seafood products, demonstrating low to total inhibition against *Listeria* sp. and low to medium activity against *E. coli.* High intraspecies antagonistic capacity of *C. maltaromaticum* was recently reported by [70]. This species is characterized by high genetic diversity [71] and is known to produce anti-listerial bacteriocins [69].

An array of characteristics of *C. maltaromaticum* and *C. divergens* are well studied, such as antilisterial activity [72,73,74,75], salt tolerance [76], adaptation to temperature changes [77], but also the possibility to spoil food [69,78] and to produce tyramine from tyrosine [69]. Thus, the genus *Carnobacterium* holds strong candidates for biopreservation, but further research is required to exclude species with spoilage potential due to inter- and intraspecies metabolic diversity [69].

Among *Leuconostoc* sp., several strains (Le.m.68, Le.l.358, Le.g.406, and Le.c.105) showed total inhibition or medium antimicrobial activity against all pathogenic strains. It should be emphasized that both *Carnobacterium* and *Leuconostoc* strains displayed medium activity against *E. coli*, which we consider a promising potential, as only a few studies describe LAB as active against Gram-negative bacteria [79,80,81,82,83]. Bacteriocins from Gram-positive bacteria are generally more effective towards Gram-positive bacteria [84] than Gram-negative bacteria, as the outer membrane (OM) of the latter makes penetration into the cell difficult [85]. Some studies have, however, shown that certain bacteriocins from Gram-positive bacteria can inhibit Gram-negative bacteria in the presence of other hurdles that weaken the OM, such as lactoferrin [86], EDTA [85], high hydrostatic pressure [87], heat treatment, and pH [88,89]. Studies have also indicated that Gram-negative bacteria can be inhibited by production of low-molecular antimicrobial products, such as lactic acid, acetic acid, carbon dioxide, diacetyl, reuterin, alcohol, and hydrogen peroxide [90,91,92,93]. Indeed, bacteriocin-negative LAB have been suggested for application of biopreservation in the seafood industry due to the occurrence of bacteriocin-resistant *L. monocytogenes* strains [69].

In previous studies *Enterococcus* sp. have shown antimicrobial activity against foodborne pathogenic microorganisms [81,94,95]. However, in our study, *Enterococcus* sp. did not display noticeable antimicrobial activity. The application of *Enterococcus* sp. in biopreservation is still controversial due to the possible production of biogenic amines (BA) [96,97].

With a wide range of medium antagonistic activity showed in our study (3.0–5.7 log CFU/mL inhibition), species from the *Weissella* genus are potential candidates for biopreservation. However, species of *Weissella* are not generally recognized as safe (GRAS), due to a possible link to human disease [98], BA production, and resistance to antibiotics [99]. These limitations are, however, strain dependent, and further research is required to elucidate their status.

Regarding the genus *Lactobacillus*, Sahnouni, et al. [100] recorded higher antimicrobial activity of *Lactobacillus* sp. compared to *Carnobacterium* sp. against pathogenic bacteria commonly reported in RTE and minimally preserved seafood products. Interestingly, *Lactobacillus* sp. did not possess significant inhibition of the targets in our study. The genus showed low or no antimicrobial activity, regardless of the source, except Lb.s.44, which showed medium activity against *L. innocua*.

## 5. Conclusions

The diversity and antimicrobial activity of LAB from a selection of RTE seafood (CSS, gravlax, and sushi) were significantly affected by the seafood source, demonstrating the importance of screening for food-derived strains for application in RTE seafood systems. Overall, the isolated LAB strains were significantly more active against *Listeria* sp. than against *E. coli* (*p* < 0.05). Against *Listeria* sp., total inhibition was observed for LAB isolated from all three seafood products. Medium antimicrobial activity (3–6 log CFU reduction) was found against *E. coli*. The observed intergenus differences in antimicrobial activity suggests that strains belonging to *Carnobacterium* spp., *Leuconostoc* spp., and *Weissella* spp. have potential to be applied as preservative cultures of minimally or lightly preserved RTE seafood, and isolates with medium to total inhibition of at least one target will be selected for future studies conducted at 4 °C or lower.

## Figures and Tables

**Table 1:** Number (percentage) of different genera of lactic acid bacteria (LAB) sampled from sushi, cold-smoked salmon (CSS), and gravlax (GL).

Seafood Source of Isolation	*n*	*Carnobacterium*	*Enterococcus*	*Lactobacillus*	*Leuconostoc*	*Weissella*
Sushi	40	10 (25)	ND	4 (10)	22 (55)	4 (10)
CSS	30	17 (57)	1 (3)	11 (37)	1 (3)	ND
GL	20	7 (35)	ND	11 (55)	1 (5)	1 (5)
Total prevalence	90 *	34 (38)	1 (1)	26 (29)	24 (26)	5 (6)

* Two strains from sushi (346) and GL (257) were not identified. ND = not detected.

## Supplementary Materials

The following are available at https://www.mdpi.com/2304-8158/10/2/271/s1, Table S1: LAB isolated from CSS (*n* = 30), gravlax (*n* = 21), and sushi (*n* = 41) against *L. innocua*, the *L. monocytogenes* (L.m.) reference strain, the *L. monocytogenes* environmental strain, and *E. coli*.
