# Supplementary material for: Diversity and Antimicrobial Activity towards Listeria spp. and Escherichia coli among Lactic Acid Bacteria Isolated from Ready-to-Eat Seafood
Source: Foods. 2021 Jan 29;10(2):271. doi: 10.3390/foods10020271 (PMC7911464; doi:10.3390/foods10020271)
Supplement: Supplementary file 1 [file foods-10-00271-s001.pdf]

Table S1: LAB isolated from CSS (n = 30), gravlax (n = 21) and sushi (n = 41) against *L. innocua, L. monocytogenes* (L.m.) reference strain., *L. monocytogenes* environmental strain and *E. coli.* Inhibition levels given as Δ log CFU/ml ± SD, i.e. difference between average concentration (log CFU/ml) of target monoculture (n = 8) and concentration (log CFU/ml) of target obtained in coculture with LAB (n=4). Different LAB isolates are marked with different letters plus isolate number (C.d. = *Carnobacterium divergens*, C.m. = *C. maltaromaticum*, Lb.s. = *Lactobacillus sakei*, Lb.f. = *Lb. fuchuensis*, Lb.c. = *Lb. curvatus*, Le.c. = *Leuconostoc citreum*, Le.g. = *L. gelidum*, Le.l. = *L. lactis*, Le.m. = *L. mesenteroides*, W.h. = *Weissella hellenica*, W.s. = *W. soli*, W.v. = *W. viridenscens*, E.p. = *Enterococcus pseudoavium*). Strains given as number only are not identified on the genus level

	LAB ID	*E. coli*	±SD	*L. innocua*	±SD	*L. m.* Ref.	±SD	*L. m.* env.	±SD
SUSHI	Lb-140	0.38	0.07	0.70	0.28	1.09	0.16	0.08	0.27
	Lb-396	-0.26	0.25	0.65	0.30	1.74	0.79	-0.08	0.25
	Lb-408	0.60	0.19	0.69	0.11	2.17	1.04	0.38	0.15
	Lb-434	0.24	0.03	0.47	0.13	0.90	0.34	1.02	0.58
	C-74	1.69	0.17	0.47	0.25	1.44	0.19	2.39	0.12
	C-153	2.45	0.16	0.12	0.19	3.53	0.52	3.11	0.26
	C-199	2.55	0.15	0.77	0.03	2.44	0.26	1.86	0.50
	C-202	0.83	0.07	2.93	0.16	2.23	0.12	1.95	0.26
	C-204	0.57	0.12	0.64	0.13	2.73	0.15	2.53	0.09
	C-316	1.70	0.14	9.38	0.00	9.11	0.00	9.39	0.00
	C-426	3.08	0.65	2.00	0.22	1.86	0.25	1.96	0.16
	C-461	3.86	0.23	9.38	0.00	9.11	0.00	9.39	0.00
	C-466	2.19	0.10	9.38	0.00	2.07	0.09	2.49	0.09
	C-468	3.13	0.41	0.46	0.20	9.11	0.00	9.39	0.00
	Leuc-63	5.15	0.16	3.95	0.18	3.80	0.35	3.95	0.40
	Leuc-67	4.89	0.13	3.92	0.20	4.01	0.06	4.41	0.20
	Leuc-68	4.59	0.24	3.91	0.33	4.07	0.07	9.39	0.00
	Leuc-88	3.91	0.24	3.64	0.00	3.55	0.25	6.51	3.32
	Leuc-126	4.49	0.34	4.08	0.54	4.07	0.09	4.34	0.08
	Leuc-151	4.19	0.17	4.04	0.21	4.29	0.05	3.48	0.06
	Leuc-152	4.41	0.16	4.27	0.07	4.34	0.18	4.60	0.13
	Leuc-292	3.39	0.09	3.42	0.16	3.08	0.22	3.87	0.39
	Leuc-294	3.89	0.32	3.63	0.30	3.22	0.10	3.54	0.16
	Leuc-298	3.49	0.14	3.33	0.05	3.02	0.30	3.73	0.18
	Leuc-299	4.76	0.11	3.52	0.33	4.39	0.20	3.94	0.16
	Leuc-301	3.61	0.35	4.19	0.41	3.59	0.38	3.87	0.10
	Leuc-340	4.18	0.22	3.30	0.32	3.24	0.13	3.75	0.21
	Leuc-342	4.13	0.15	3.93	0.34	3.61	0.05	3.42	0.31
	Leuc-345	4.24	0.11	3.59	0.68	3.05	0.21	3.64	0.18
	Leuc-357	3.46	0.31	3.41	0.23	3.43	0.22	3.56	0.16
	Leuc-358	4.48	0.38	1.96	0.26	9.11	0.00	5.02	0.33
	Leuc-361	3.47	0.29	4.20	0.58	3.14	0.34	3.36	0.08
	Leuc-405	4.24	0.09	4.05	0.35	3.90	0.29	3.60	0.11
	Leuc-406	4.63	0.26	9.38	0.00	9.11	0.00	9.39	0.00

	Leuc-455	3.76	0.29	4.41	0.39	4.02	0.08	4.26	0.48
	Leuc-457	3.79	0.26	3.37	0.11	3.43	0.11	3.92	0.07
	W-198	0.31	0.12	3.09	0.37	3.01	0.43	0.58	0.11
	W-321	2.11	0.48	3.29	0.22	1.81	0.73	2.60	0.12
	W-327	4.49	1.15	4.78	0.23	4.96	0.70	5.70	0.37
	W-328	4.59	0.09	5.17	0.42	4.68	0.20	5.25	0.09
	346	4.00	0.09	3.02	0.49	3.28	0.17	3.56	0.93
CSS	Lb-1	1.06	0.95	-0.23	0.36	0.77	0.42	0.25	0.29
	Lb-7	0.10	0.21	-0.51	0.15	0.84	0.45	0.23	0.12
	Lb- 44	0.46	0.21	0.73	0.13	1.18	0.09	6.87	3.03
	Lb- 90	1.47	0.48	0.31	0.40	1.68	0.16	0.41	0.23
	Lb- 221	0.36	0.14	1.46	0.66	0.75	0.10	0.14	0.14
	Lb- 258	0.17	0.10	2.62	0.27	0.25	0.21	-0.56	0.09
	Lb- 260	0.29	0.11	0.46	0.14	1.34	0.50	0.44	0.84
	Lb- 303	0.04	0.37	-0.01	0.10	0.96	0.10	0.15	0.22
	Lb- 305	0.13	0.11	1.53	0.14	0.46	0.31	0.09	0.09
	Lb- 388	-0.23	0.24	0.45	0.06	0.57	0.11	0.66	0.27
	Lb- 443	0.12	0.07	0.03	0.29	0.88	0.27	0.22	0.30
	C-6	3.46	0.10	0.71	0.15	3.27	0.14	3.05	0.31
	C- 8	1.52	0.28	0.65	0.11	1.55	0.05	1.58	0.14
	C-12	0.64	0.12	3.31	0.58	2.48	0.27	2.52	0.14
	C-13	3.06	0.11	5.66	0.12	9.11	0.00	9.39	0.00
	C-14	0.63	0.07	0.55	0.11	1.28	0.16	0.90	0.33
	C-21	3.95	0.70	3.03	0.26	3.35	0.67	1.53	1.05
	C-42	1.82	0.11	9.38	0.00	9.11	0.00	9.39	0.00
	C-55	3.11	0.46	9.38	0.00	9.11	0.00	9.39	0.00
	C-60	0.87	0.10	1.05	0.26	2.88	0.21	2.67	0.14
	C-95	2.32	0.22	0.58	0.12	2.46	0.15	2.50	0.13
	C-159	1.43	0.13	9.38	0.00	9.11	0.00	9.39	0.00
	C-176	0.94	0.58	0.06	0.10	1.06	0.04	0.79	0.29
	C-208	2.19	0.10	1.35	0.20	1.56	0.09	0.96	0.31
	C-214	3.09	0.21	0.59	0.11	2.43	0.22	2.95	0.25
	C-368	-0.83	0.08	0.69	0.12	2.32	0.06	2.77	0.10
	C-384	2.69	0.11	1.07	0.11	3.02	0.35	2.83	0.14
	C-392	1.54	0.21	0.08	0.30	1.64	0.42	1.17	0.43
	Leuc-93	4.38	0.14	4.12	0.11	3.68	0.16	4.62	0.18
	Ent-259	2.73	0.12	2.62	0.27	3.02	0.24	2.44	0.14
GRAVLAX	Lb-36	0.30	0.07	0.00	0.12	0.69	0.26	0.29	0.03
	Lb-38	0.21	0.22	0.58	0.06	0.65	0.38	0.05	0.13
	Lb-228	0.19	0.20	0.44	0.03	1.36	0.24	-0.04	0.15
	Lb-232	0.08	0.18	0.76	0.05	0.05	0.60	0.43	0.11
	Lb-237	0.54	0.35	0.79	0.46	0.75	0.34	0.03	0.39
	Lb-238	0.17	0.10	0.13	0.23	0.90	0.26	0.56	0.12
	Lb-265	0.28	0.13	0.92	0.39	0.90	0.20	0.20	0.29
	Lb-306	0.13	0.09	0.59	0.78	0.32	0.03	0.41	0.45

	Lb-309	-0.15	0.16	0.42	0.07	0.18	0.08	0.53	0.22
	Lb-420	0.31	0.24	0.40	0.16	1.10	0.17	0.32	0.06
	Lb-423	0.20	0.15	0.43	0.11	0.65	0.14	0.53	0.25
	C-27	2.14	0.10	3.78	0.30	3.55	0.16	3.12	0.05
	C-30	1.66	0.29	9.38	0.00	9.11	0.00	9.39	0.00
	C-35	2.05	0.24	9.38	0.00	9.11	0.00	9.39	0.00
	C-113	2.64	0.79	1.86	0.28	0.53	0.09	1.55	0.24
	C-227	3.16	0.22	9.38	0.00	3.19	0.07	2.89	0.19
	C-236	0.73	0.06	2.88	0.20	2.24	0.36	2.02	0.32
	C-273	3.70	0.30	2.01	0.44	1.91	0.04	2.72	0.18
	Leuc-105	3.13	0.15	7.73	1.91	9.11	0.00	9.39	0.00
	W-312	3.35	0.20	4.79	0.41	4.34	0.27	1.83	0.57
	257	0.10	0.44	0.34	0.18	1.07	0.12	1.81	0.05
